# Preferential helping to relatives: A potential mechanism responsible for lower yield of crop variety mixtures?

**DOI:** 10.1111/eva.12842

**Published:** 2019-08-01

**Authors:** Hélène Fréville, Pierre Roumet, Nicolas Olivier Rode, Aline Rocher, Muriel Latreille, Marie‐Hélène Muller, Jacques David

**Affiliations:** ^1^ AGAP, Univ Montpellier, CIRAD, INRA, Montpellier SupAgro Montpellier France; ^2^ CBGP, Univ Montpellier, CIRAD, Montpellier SupAgro, INRA IRD Montpellier France

**Keywords:** agriculture, durum wheat, indirect genetic effect, kin recognition, kin selection, phenotypic plasticity, plant height

## Abstract

Variety mixtures, the cultivation of different genotypes within a field, have been proposed as a way to increase within‐crop diversity, allowing the development of more sustainable agricultural systems with reduced environmental costs. Although mixtures have often been shown to over‐yield the average of component varieties in pure stands, decreased yields in mixtures have also been documented. Kin selection may explain such pattern, whenever plants direct helping behaviors preferentially toward relatives and thus experience stronger competition when grown with less related neighbors, lowering seed production of mixtures. Using varieties of durum wheat originating from traditional Moroccan agrosystems, we designed a greenhouse experiment to address whether plants reduced competition for light by limiting stem elongation when growing with kin and whether such phenotypic response resulted in higher yield of kin groups. Seeds were sown in groups of siblings and nonkin, each group containing a focal plant surrounded by four neighbors. At the group level, mean plant height and yield did not depend upon relatedness among competing plants. At the individual level, plant height was not affected by genetic relatedness to neighbors, after accounting for direct genetic effects that might induce among‐genotype differences in the ability to capture resources that do not depend on relatedness. Moreover, in contrast to our predictions, shorter plants had lower inclusive fitness. Phenotypic plasticity in height was very limited in response to neighbor genotypes. This suggests that human selection in crops may have attenuated shade‐avoidance responses to competition for light. Future research on preferential helping to relatives in crops might thus target social traits that drive competition for other resources than light. Overall, our study illustrates the relevance of tackling agricultural issues from an evolutionary standpoint and calls for extending such approaches to a larger set of crop species.

## 1 INTRODUCTION

Social interactions among conspecifics are common in a wide range of species including microorganisms, animals, and plants (Frank, 1998). Traits involved in social interactions, also called “social traits”, are those for which the phenotype of an individual affects both the performance of that individual and the performance of other individuals in interaction (Hamilton, 1964a). Social interactions thus lead to multilevel selection operating both at the individual and at the group levels (Lehmann, Keller, West, & Roze, 2007; Lion, Jansen, & Day, 2011; Wade, 1977). Kin selection theory provides a relevant framework to understand the evolution of social traits. This theory predicts that a phenotype that decreases individual's performance can be favored if the performance of some other interacting individuals is increased by the focal individual's phenotype, and if these “recipient” individuals are genetically related to the focal individual, resulting in an increase in inclusive fitness (Hamilton, 1964a; Maynard Smith, 1964). Kin selection theory has been very successful in explaining the evolution of helping behaviors including altruistic behaviors, whereby the actor pays a cost on its direct fitness but increases inclusive fitness (Hamilton, 1964a).

In agriculture, it is well established that the phenotype of a plant affects the fitness of its neighbors (Donald & Hamblin, 1983; Jensen & Federer, 1964). During the Green Revolution, this led to the definition of the “weak competitor” crop ideotype, and the breeding of plants with phenotypic characteristics such as short stems and few erect small leaves in wheat (Donald, 1968). Thus, cultivated plants experience social interactions with conspecifics within fields. In seed crops, farmers have not only selected at the individual level by picking plants that fitted best with their phenotypic criteria. Indeed, yield, the seed production of the group, has always been a selection target (Donald, 1968; Donald & Hamblin, 1983). The most productive fields may thus have contributed the most to the next generation, leading to multilevel selection. In this context, the existence of kin recognition mechanisms that allow helping behaviors to be directed preferentially toward kin (Hamilton, 1964b; Lehmann & Perrin, 2002) may have facilitated the evolution of helping behavior in seed crops when selecting for yield.

Variety mixtures, that is, the cultivation of different genotypes within a field, have been proposed as a way to take better advantage of ecological processes naturally occurring in ecosystems allowing the development of more sustainable agricultural systems with reduced environmental costs (Barot et al., 2017; Hajjar, Jarvis, & Gemmill‐Herren, 2008; Østergård et al., 2009). For instance, the resource‐use complementarity hypothesis predicts that mixtures of genotypes that differ in resource‐related traits will make better use of the total amount of available resources (Macarthur & Levins, 1967). Accordingly, variety mixtures have been shown to over‐yield the average of component varieties in pure stands in many crops (wheat, rice, barley, oat, maize, sorghum, etc; Kiær, Skovgaard, & Østergård, 2009; Reiss & Drinkwater, 2018; Smithson & Lenné, 1996), suggesting negative effects of genetic relatedness on the outcome of competition (see Barot et al., 2017 for a review of possible mechanisms at play in mixtures). Variety mixtures may thus represent a relevant strategy to promote increased yield with minimal environmental impact, particularly for crops such as wheat, rice, and barley, which have been cultivated in monogenotypic cultures of pure lines in intensive agrosystems. Still, decreased yields in mixtures compared to monocultures have also been documented in these same species (Kiær et al., 2009; Smithson & Lenné, 1996), suggesting positive effects of genetic relatedness on the outcome of competition. Preferential helping of relatives might explain why monocultures sometimes outperform mixtures. This hypothesis implies that individuals show a plastic response to relatedness of the neighbors on some social traits and that such phenotypic responses lead to reduced competition among kin, resulting in higher inclusive fitness in kin groups and thus higher yield in monocultures (File, Murphy, & Dudley, 2012; Hamilton, 1964b; Lehmann & Perrin, 2002). Preferential helping of relatives requires kin recognition mechanisms (Hamilton, 1964b; Rousset & Roze, 2007). Assuming genetic variation among varieties at the loci involved in kin recognition, we expect helping behaviors to occur less frequently within mixtures than within pure stands. Kin recognition mechanisms in crops may explain the observed discrepancy of results among studies of variety mixtures within species, the outcome of competition among plants depending upon the genetic composition of the chosen varieties at the kin recognition loci.

Although historically studied in animals and microorganisms (Crespi, 2001; Hamilton, 1964b), preferential helping of relatives has received increased attention in plants in recent decades (Dudley & File, 2007; File et al., 2012; Masclaux et al., 2010; Milla, Forero, Escudero, & Iriondo, 2009; Murphy & Dudley, 2009). Some authors have suggested that plants can recognize their kin and display phenotypic plasticity toward reduced competition for resources when growing with kin as expected under kin selection (Dudley & File, 2007; Dudley, Murphy, & File, 2013; File et al., 2012). For instance, the reduced biomass of fine roots of plants of *Cakile edentula* grown with kin compared to nonkin has been interpreted as the result of competition avoidance for resources among kin (Bhatt, Khandelwal, & Dudley, 2011; Dudley & File, 2007). Yet in the study of Dudley and File (2007) where fitness was estimated, phenotypic responses to relatedness did not entail increased yield in kin groups, questioning the interpretation of reduced competition for resources among kin. More recently, Crepy and Casal (2015) showed that plants of *Arabidopsis thaliana* reduced mutual shading by reorienting leaf growth when grown with kin, leading to higher yield compared to kin groups of mutants unable to reorient leaf growth. Still, kin groups of the wild type had lower yield than nonkin groups. To our knowledge, preferential helping of relatives has never been proposed as a possible mechanism to explain reduced performance of variety mixtures.

The occurrence of preferential helping of relatives remains controversial in plants since the pioneer work of Dudley and File (2007) (see, for instance, Klemens, 2008; Till‐Bottraud & de Villemereuil, 2016). First, differential phenotypic responses to relatedness of the neighbors have often been interpreted as preferential helping. Yet, such phenotypic changes can be caused by several mechanisms, including niche complementarity arising from plasticity on resource‐related traits (File et al., 2012; Milla et al., 2009). Second, the outcome of competition at the group level has been shown to depend upon the choice of genotypes grown together (Cheplick & Kane, 2004; Crepy & Casal, 2015; Masclaux et al., 2010), casting doubts on the many studies that do not control for genotype effects. Genetic variations on social traits involved in resource competition lead to among‐genotype differences in the ability to capture resources that do not involve relatedness, and can thus hamper the detection of preferential helping of relatives (Masclaux et al., 2010).

Identifying relevant social traits is the key to test for preferential helping of relatives. Still, such traits are poorly known in plants. Social traits in plants have mainly been discussed in the context of the tragedy of the commons (TOC), whereby competition for resources drives the evolution of traits toward phenotypic values lowering group performance (Anten & Vermeulen, 2016; Hardin, 1968). Such traits involved in resource acquisition (see Anten & Vermeulen, 2016 for a list of traits for which a TOC has been documented) are good candidates because we might expect plastic responses to relatedness on these traits to reflect changes in the strength of competition for resources and thus an effect on group performance. When plants compete for light, height certainly represents the most emblematic example of traits that lead to a TOC (Anten & Vermeulen, 2016): tall plants win access to light over shorter plants, but incur direct costs in construction and maintenance of vegetative structures leading to a negative correlation between plant height and seed production of the group (Anten & Vermeulen, 2016; Falster & Westoby, 2003). This pattern has been well documented in crops (Austin et al., 1980; Donald, 1963; Jennings & Aquino, 1968). Multilevel selection analyses performed in species where plants compete for light confirm this view: individual and group selection on height operate in opposite directions, with selection at the group level favoring small plants and selection at the individual level favoring tall plants (Kelly, 1996; Stevens, Goodnight, & Kalisz, 1995). Thus, for species competing for light, being short might be considered as an altruistic behavior. Assuming that height is involved in preferential helping of relatives, we thus expect plants to exhibit smaller stem elongation when growing with kin compared to nonkin (Biernaskie, 2011; Dudley & File, 2007), and plant height and inclusive fitness to be negatively correlated.

Here, we set up a pot experiment with either monogenotypic groups or mixtures of two genotypes to study the effects of relatedness on plant height and yield in durum wheat, a major staple crop for which cultivation of genotype mixtures might help reducing chemical inputs and improve disease control. First, we tested whether the mean allocation to height in monogenotypic groups was lower than in mixtures and resulted in higher yield, as expected under preferential helping of relatives involving plant height. Second, we used a quantitative genetic model to test whether height measured at the individual level was lower in monogenotypic groups after accounting for potential variations in main direct genetic effects in plant height, which might lead to among‐genotype differences in the ability to capture resources that do not depend on relatedness. Third, we tested whether there was a negative relationship between plant height and inclusive fitness, after accounting for genotype effects.

## 2 MATERIALS AND METHODS

### 2.1 Study species

Durum wheat, *Triticum turgidum* ssp. *durum* (Desf.) MacKey, is a staple crop, mostly used for the production of pasta and semolina. It is derived from the wild emmer *Triticum turgidum* ssp. *dicoccoides* (körn.) Thell and was among the first crop to be domesticated in the Fertile Crescent ~10,000 years ago (Salamini, Ozkan, Brandolini, Schafer‐Pregl, & Martin, 2002). Durum wheat is a highly selfing species: its selfing rate is being considered higher than 95% by breeders and has been estimated between 97% and 99% in Ethiopian landraces (Tsegaye, 1996). In modern agricultural systems, durum wheat is grown in monogenotypic cultures of pure lines. Thus, no genetic diversity is present at the field level. In some regions, durum wheat cultivation is nowadays jeopardized by its high environmental and economic costs due to intensive use of fertilizers and by recurrent breakdown of genetically controlled resistance to pathogens. Moreover, the narrow genetic diversity of the elite line pool (Haudry et al., 2007; Thuillet, Bataillon, Poirier, Santoni, & David, 2005) and strong genetic trade‐offs among bred traits (e.g., yield and protein content) have hampered genetic progress and the development of innovative varieties adapted to more sustainable agrosystems. In this context, genotype mixtures might help maintaining high quality and quantity production while reducing chemical inputs, provided that preferential helping to relatives does not lead to increased competition among plants of different genotypes, and an accumulation of biomass in the vegetative parts to the detriment of seed production.

### 2.2 Seed origin

Plant material originated from a large‐scale survey of Moroccan traditional durum wheat varieties conducted by Chentoufi et al. (2014). In contrast to modern durum wheat agrosystems, traditional Moroccan agrosystems still harbor genetic diversity, among fields (Muller et al in prep), among individuals within field (this study, Muller et al in prep), and within individuals (Sahri et al., 2014), making it more likely to find polymorphism at kin recognition loci. In addition, if kin selection has been acting on durum wheat phenotypes, the maintenance of genetic diversity within fields could have favored the maintenance of a kin recognition system (Hamilton, 1964b). We thus expect plants from traditional farming systems to be particularly relevant to test for the existence of preferential helping to relatives. Among the 166 fields surveyed by Chentoufi et al. (2014), we selected two fields in the village of Anfergual (latitude: 32.299444; longitude: −5.059166) and two fields in the village of Tiydrine (latitude: 32.274166; longitude: −4.908333) (see Chentoufi et al., 2014 for details). These villages are located in the Atlas Mountains in a region with the highest durum wheat genetic diversity (Area ZS2 in Sahri et al., 2014). They are 15 km apart and are characterized by similar ecological conditions. Seeds from 20 different maternal plants randomly sampled in each field were grown in an experimental field in Montpellier, southern France (latitude: 43.618679; longitude: 3.855386), from March to July 2014. These plants were self‐fertilized for one generation to produce the material used in this study. In the following, individuals descending from a single selfed maternal plant are referred to as a “family”. This protocol allowed us to remove any maternal effect that may contribute to differences in growth and reproductive success among families. In addition, one seed from each of the 80 original maternal plants was sown and genotyped using 26 nuclear microsatellite loci described in Table S1. For each field, 13 of the 20 maternal plants were finally kept after excluding those with heterozygous loci, and those which progenies gave insufficient number of selfed seeds for conducting our experimental design. The 52 chosen maternal plants represented unique multilocus genotypes. Because of the high rate of selfing of durum wheat and the exclusion of heterozygous maternal plants, we expect within‐family relatedness to be very high compared to among‐family relatedness. In addition, mixtures of fixed varieties have been the most straightforward way to increase within‐crop genetic diversity in the field. The use of highly inbred genotypes is thus highly relevant to test our hypothesis. For the statistical analysis, we considered that plants belonging to the same family had the same multilocus genotype.

### 2.3 Experimental design

We conducted two experiments in a greenhouse located at the French National Institute for Agricultural Research in Mauguio, southern France (Experimental Unit DiasCope, latitude: 43.610406; longitude: 3.977511), from March to July 2015.

#### 2.3.1 Competition experiment

This experiment was conducted first to test whether growing with four neighbors as in the kin experiment induced competition among plants sharing a pot and second to assess whether plant height showed phenotypic plasticity in response to the number of neighbors. Of the 52 families used in this study, 32 (8 per field) were randomly chosen. Each family was grown in two adjacent pots, one with five plants sown as in the kin experiment (see below), and one with only one plant located in the center of the pot. This experiment contained 64 pots placed on a separate shelve and a total of 192 plants. Location of pairs of family pots was randomized.

#### 2.3.2 Kin experiment

This experiment aimed at testing the effects of relatedness on plant height and yield in durum wheat. Each pot contained five plants, with a focal plant placed in the center of a diamond formed by four neighboring plants. The focal seedling and its neighbors were sown 4 cm apart from each other. Each pot was assigned to one of two relatedness treatments: (a) kin treatment (seeds belonging to the same family) and (b) nonkin treatment (focal plant and neighbors belonging to two different families). Pots were arranged in two tables (blocks) in the greenhouse. Each block was divided into sub‐blocks, each sub‐block containing 8 pots with the same family used for focal plants. Each of the 52 families was randomly assigned to a block and a sub‐block. For each family used as a focal, the within sub‐block design incorporated the kin treatment (2 pots) and the nonkin treatment (6 pots, each with a different neighbor family). We did not replicate combinations of genotypes within the nonkin treatment. Indeed, the nonkin effect was more accurately estimated by averaging values of response variables over multiple different combinations with one replicate rather than over few combinations with many replicates. Each family was represented six times as a neighbor when used in the nonkin treatment. Pairs of competing families were drawn randomly. Location of pots within each sub‐block was randomized. This experiment contained 416 pots and a total of 2080 plants. As expected, genetic relatedness calculated as the proportion of shared alleles between the focal family and the neighbor family (Lynch, 1988) was significantly lower in the nonkin treatment than in the kin treatment (54.8% vs. 100%) (Wilcoxon's signed‐rank test: W = 32,448, *p* < .001). The proportion of shared alleles between families ranged from 11.6% to 88.4% within the nonkin treatment, whereas it was equal to 100% within the kin treatment (Figure S1). Kin recognition mechanisms lead to individuals behaving toward each other according to genetic similarity that arises from common ancestry (Grafen, 1990; Hamilton, 1964b). Relatedness was included in statistical analyses using two proxies of genetic similarity. First, genetic similarity was included as a categorical variable reflecting common ancestry as described above (kin, nonkin). Second, genetic similarity was calculated as the proportion of shared alleles between the focal plant and its neighbors and was thus included as a continuous variable in the models.

### 2.4 Growing conditions

Seeds from the 52 families were weighed individually (we hereafter refer to this variable as initial seed mass). On March 06, 2015, they were placed individually onto moistened filter paper in petri dishes stored at 4°C for 1.5 days and then transferred to a growth chamber (20°C) for 1.5 days. Three‐day‐old seedlings were transplanted to plastic pots (1L, 20 cm diameter) containing soil medium (80% compost and 20% sand). Twelve additional seeds per family were individually weighed and grown as supplements in case of casualties during the first 10 days of the experiment. Thirty seeds were replaced out of 2,272 (two in the competition experiment and 28 in the kin recognition experiment). Pots were regularly watered and complemented with N‐P‐K fertilizers to limit the effects of competition for soil resources compared to competition for light.

### 2.5 Plant measurements

The 2,272 plants from both experiments were measured and harvested between July 9 and July 17, 2015. To assess whether a plant with four neighbors experienced competition compared to plants grown individually, we recorded the number of tillers and estimated vegetative biomass, two traits negatively impacted by competition in annual seed crops (Donald, 1963; Jennings & Aquino, 1968). The number of tillers was obtained by summing reproductive tillers and regressed tillers that remain vegetative. The aboveground part of each plant was clipped manually at the soil surface and partitioned into spikes and shoots. Aerial parts were dried, and shoots were weighed to obtain vegetative biomass. Plant height was measured as the length of the tallest tiller recorded from the ground to the tip of the spikes excluding awns. Spikes were threshed to retrieve fully developed seeds and aborted seeds. Both types of seeds were counted and weighed automatically using the technology “Optoagrimetric” developed by Optomachines (http://optomachines.fr). We estimated yield by summing the number of fully developed seeds over the five plants within pots. Central to the kin selection theory is the concept of inclusive fitness (Hamilton, 1964a), in which estimation has led to confusion in the empirical literature (Grafen, 1982). Following Grafen (1982), we estimated inclusive fitness as the number of fully developed seeds of the focal individual. This measure accounts for the influence of social partners on reproductive success and thus includes kinship effects (Grafen, 1982).

### 2.6 Data analysis

#### 2.6.1 Competition experiment

We tested for the effect of the number of neighbors per pot (0 or 4) on the number of tillers, vegetative biomass, fitness, and height of the focal plant, using generalized linear mixed models (GLMMs) with genotype as a random‐effect factor and the number of plants per pot as a fixed‐effect factor. Initial seed mass data of focal plants were Z‐standardized and were included as a covariate. Vegetative biomass and plant height were analyzed using a linear model with a normally distributed error. The number of tillers and plant fitness were analyzed using a Poisson error distribution with a log link function. Significance of fixed effects was assessed by computing 95% confidence intervals on the model parameters from the likelihood profile (Bates, Machler, Bolker, & Walker, 2015). GLMM analyses were performed using the lme4 package in R (Bates et al., 2015).

#### 2.6.2 Kin experiment

##### 2.6.2.1 Group‐level analyses

To test for an effect of relatedness on mean height and yield, we used the following linear mixed model:

(1) $$Y_{\text{group}}=Xb+Zu+\varepsilon$$

where *Y*_group_ denotes the vector of 416 observations at the group level (mean height or yield); *b* is a vector of fixed effects including block, initial seed mass, and relatedness; *X* is the 416 × 3 design matrix that associates observations with the appropriate combination of the three fixed effects; u is a vector of random genetic effect accounting for the genotypic composition of the group made of either 5 plants having the same genotype (kin treatment) or five plants with one having a different genotype from the four others (nonkin treatment); Z is a 416 × 52 matrix relating observations to the appropriate combinations of random effects, each of the 52 genotypes being represented 0, 1, 4 or 5 times within a pot; and *ε* is the vector of residual errors. We thus assumed that a genotype had the same effect on yield whatever its position in the pot. Random effects in *u* were assumed to be normally distributed with mean zero and variance *σ* ^2^. For each pot, initial seed mass was calculated as the sum of individual initial seed mass over the five plants. Seed mass data and data of genetic similarity measured as the proportion of shared alleles between the focal plant and its neighbors were Z‐standardized. To account for potential environmental effects occurring at a finer scale within blocks, we partitioned the random error *ε* into an uncorrelated error *ε*_uncor_ by including pot as a random factor and a spatially correlated environment error *ε*_cor_ that models the effect of shared environmental conditions by adjacent pots on a grid (“AR1 two‐dimensional spatial models”) (Gilmour, Gogel, Cullis, Thompson, & Butler, 2009). Thus, if *ε*_cor_ ≠ 0 the closer the two pots are within a block, the higher the correlations are between the residual errors of the model.

##### 2.6.2.2 Individual‐level analyses

Variations in mean plant height of the group can depend upon direct genetic effects that lead to among‐genotype differences in the ability to capture light that does not depend on relatedness, and can thus hamper the detection of preferential helping to relatives (Masclaux et al., 2010). To control for this effect, we tested whether focal plant height responded to neighbor relatedness, using a quantitative genetic model that accounts for the effect of the genes of the focal individual, known as the direct genetic effect (DGE), and the effect of the genes of neighbors, known as indirect genetic effect (IGE), as follows:

(2) $$Y_{\text{focal}}=Xb+Z_{\text{f}}u_{\text{f}}+Z_{\text{n}}u_{\text{n}}+\varepsilon$$

where *Y*_focal_ denotes the vector of 416 observations of height measured on focal plants; *b* is a vector of fixed effects including block, initial seed mass, and relatedness (see Eq. 1 for notations *X* and *ε*); *u*_f_ and *u*_n_ are vectors of random genetic effects for the focal individuals (GDE) and the neighbors (IGE), respectively; and *Z*_f_ and *Z*_n_ are 416 × 52 matrices relating observations to the appropriate combinations of random effects, 52 being the number of genotypes used in the experiment. In contrast to Equation (1) that only accounts for the direct effects of the genes (DGE) of each of the five individuals within a pot, Equation (2) accounts for both DGE and IGE and thus allowed us to test for the effect of relatedness on height independently of the main direct effects of the genes of the competing genotypes. The random effects in *u*_f_ and in *u*_n_ were assumed to follow a multivariate normal distribution with a mean of zero but with different variances, $\sigma_{\text{f}}^{2}$ and $\sigma_{\text{n}}^{2}$, respectively. In addition, we included a genetic covariance term *σ*_fn_ between the direct genetic effect of the focal genotype and the indirect genetic effect of this genotype as a neighbor (Wolf, 2003); *σ*_fn_ measures the degree to which genes simultaneously affect *Y*_ind_ of focal individuals and *Y*_ind_ of their social partners. For instance, a negative value of the covariance *σ*_fn_ would indicate that genes that make an individual taller make their partner shorter. Fixed effects included block, initial seed mass, and relatedness. As in Equation (1), the random error was partitioned into an uncorrelated error by including pot as a random factor and a spatially correlated environment error. Finally, to test whether smaller stem elongation increased inclusive fitness as expected under the kin selection theory, we used a linear mixed model where inclusive fitness estimated as the number of fully developed seeds of the focal plant was added as a fixed effect in place of relatedness in Equation (2). This analysis allowed us to test for a negative relationship between plant height and inclusive fitness independently of the IGE on height due to competing genotypes.

##### 2.6.2.3 Model selection and significance of fixed effects

The relevance of including random‐effect factors was assessed by model selection based on the corrected Akaike's information criterion AIC_c_ (Burnham & Anderson, 2002). We considered that any model having an AIC_c_ difference less than two compared to the model with the lowest AIC_c_ (∆AIC_c_ < 2), while not as good as the best model, had substantial support, except when it differed from the latter by only one parameter and had a similar log‐likelihood (Burnham & Anderson, 2002, p131). Fixed effects were then tested by fitting the best model(s) and using incremental Wald *F* tests with a 5% significance level (Gilmour et al., 2009). Assumptions of linear models were checked by visual assessment of plots of the residuals against fitted values and normal quantile–quantile plots. Linear mixed‐model computations were performed using the ASReml‐R package in R (asreml 3.0, VSN International; Gilmour et al., 2009).

## 3 RESULTS

No plant mortality was observed in our experiments. Thus, all pots were included in the analyses. Results for the model selection procedure are reported in Table S2–Table S5.

### 3.1 Competition experiment

**Figure 1:**
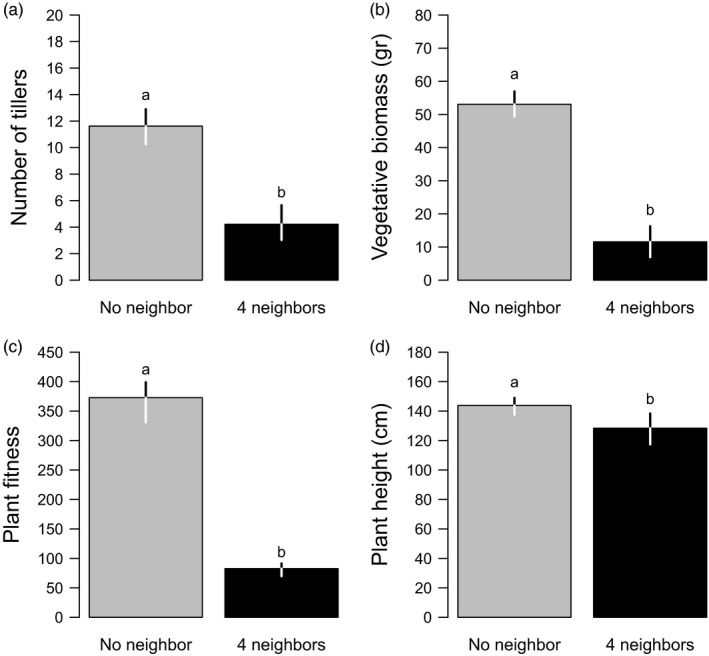
Effect of growing alone (gray bars) and surrounded by four neighbors (black bars) on (a) number of tillers, (b) vegetative biomass, (c) plant fitness, and (d) plant height, measured on the plant located in the center of the pot. Bars represent observed values. Segments represent 95% confidence intervals computed from the likelihood profile. Different letters mean significant differences between growing conditions (*p* < .05)

Plants grown with four neighbors produced a smaller number of tillers (mean _4 neighbors_ = 4, mean _no neighbor_ = 12; Figure 1a) and less vegetative biomass (mean _4 neighbors_ = 12 gr, mean _no neighbor_ = 53 gr; Figure 1b). Moreover, they had a lower fitness (mean _4 neighbors_ = 80 seeds, mean _no neighbor_ = 363 seeds; Figure 1c). This indicates that plants grown with four neighbors did experience competitive interactions. Plant height showed a plastic response to the number of neighbors, plants being shorter when grown with four neighbors (mean _4 neighbors_ = 127 cm, mean _no neighbor_ = 143 cm; Figure 1d).

### 3.2 Effects of relatedness on mean plant height and yield

**Figure 2:**
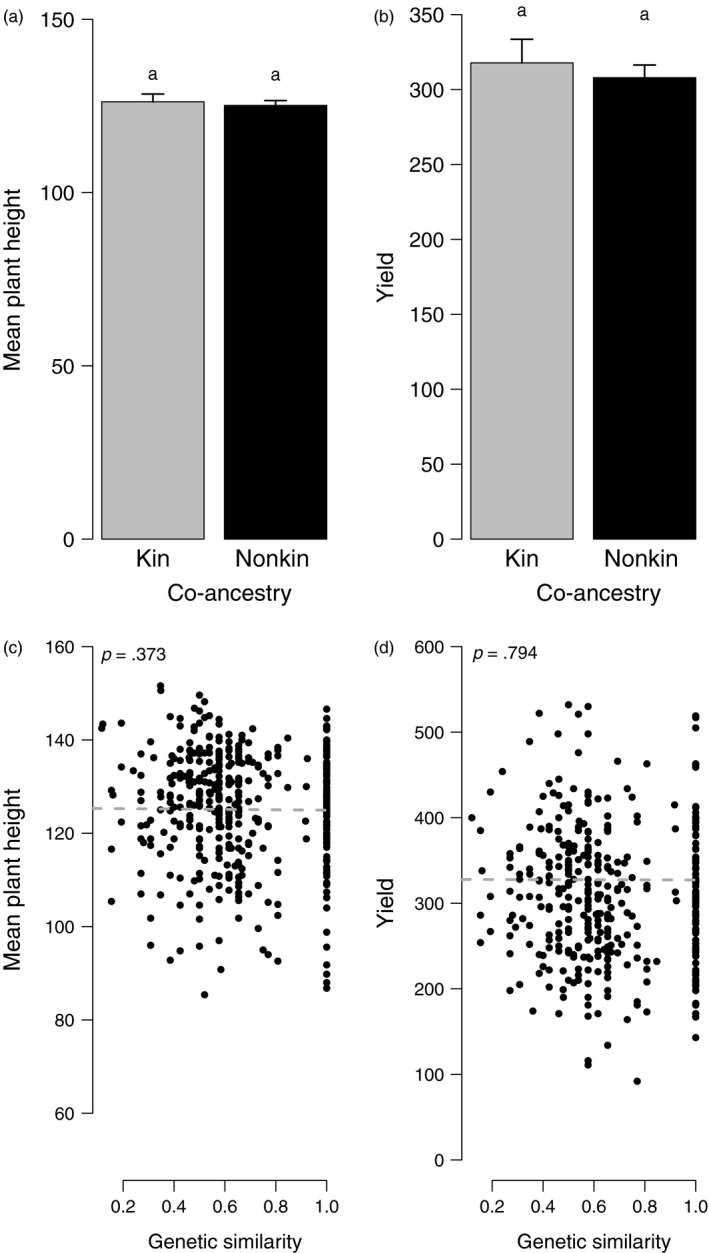
Effects of relatedness on mean plant height and yield, examined at the group level. In Figs (a) and (b), relatedness was estimated as co‐ancestry. Bars represent observed values. Segments represent standard errors computed after accounting for other effects included in the best model (Tables S2 and S3). Different letters mean significant differences between kin and nonkin (*p* < .05). In Figs (c) and (d), relatedness was estimated as genetic similarity (proportion of shared alleles between the focal genotype and the neighbor genotype). The linear relationship is plotted according to the best model (Tables S2 and S3)

There was no evidence that relatedness affected mean plant height (Table 1, Table S2) and yield (Table 2, Table S3). Mean plant height was not smaller in kin groups (mean _kin_ = 126 cm, mean _non kin_ = 125 cm; *p* = .627, Figure 2a) and did not decrease with increasing genetic similarity (*b* = −.402, *p* = .373, Figure 2c). Yield was not larger in kin groups (mean _kin_ = 318 seeds, mean _non kin_ = 308 seeds; *p* = .998, Figure 2b) and did not increase with increasing genetic similarity (*b* = −.709, *p* = .794, Figure 2d).

**Table 1:** Results from GLMMs of mean plant height of the group to relatedness

Source of variation	With co‐ancestry			With genetic similarity		
	Df	Wald F	*p*‐value	Df	Wald F	*p*‐value
Intercept	6.2	3,821.000	**<.001**	6.1	3,797.000	**<.001**
Block	5.3	0.021	.890	5.3	0.020	.892
Seed mass	118.9	7.398	**.008**	118.4	7.436	**.007**
Relatedness	320.6	0.237	.627	328.4	0.796	.373

Note

Relatedness was included either as a categorical variable “co‐ancestry” with two classes (kin and nonkin) or as a continuous variable measuring “genetic similarity” (proportion of shared alleles between the focal genotype and the neighbor genotype). Effects were tested using incremental Wald *F* tests with a 5% significance level after fitting the best model for random‐effect factors (see text for details on model construction and Table S2). The degree of freedom of the numerator was equal to 1 for all terms. We only report the degree of freedom of the denominator. *p*‐Values <.05 are in bold.

**Table 2:** Results from GLMMs of yield to relatedness

Source of variation	With co‐ancestry			With genetic similarity		
	Df	Wald F	*p*‐value	Df	Wald F	*p*‐value
Intercept	30.9	933.200	**<.001**	30.8	933.000	**<.001**
Block	5.4	1.880	.224	5.4	1.876	.225
Seed mass	144.1	4.872	**.029**	143.9	4.866	**.029**
Relatedness	266.8	< 0.001	.998	274.7	0.068	.794

Note

Relatedness was included either as a categorical variable “co‐ancestry” with two classes (kin and nonkin) or as a continuous variable measuring genetic similarity (proportion of shared alleles between the focal genotype and the neighbor genotype). Effects were tested using incremental Wald *F* tests with a 5% significance level after fitting the best model for random‐effect factors (see text for details on model construction and Table S3). The degree of freedom of the numerator was equal to 1 for all terms. We only report the degree of freedom of the denominator. *p*‐Values <.05 are in bold.

### 3.3 Effect of relatedness on focal height

**Figure 3:**
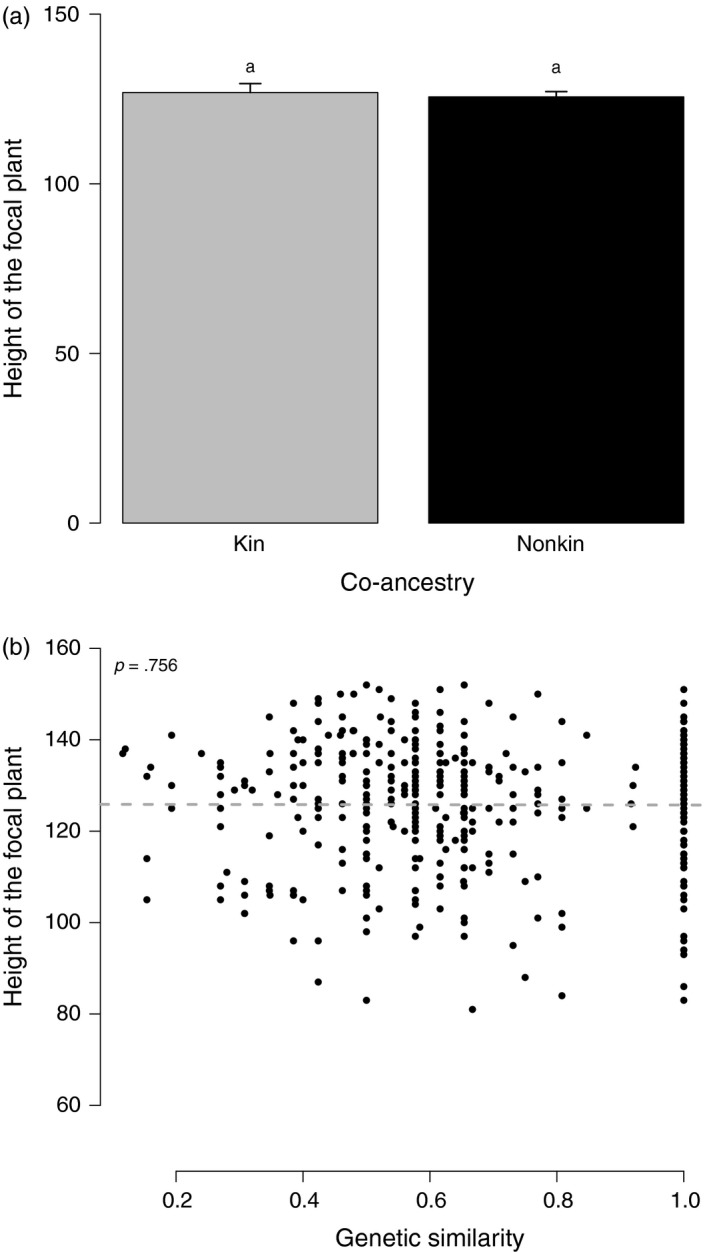
Effects of relatedness on the height of the focal plant. In (a), relatedness was estimated as co‐ancestry. Bars represent observed values. Segments represent standard errors computed after accounting for other effects included in the best model (Table S4). Different letters mean significant differences between kin and nonkin (*p* < .05). In (b), relatedness was estimated as genetic similarity (proportion of shared alleles between the focal genotype and the neighbor genotype). The linear relationship is plotted according to the best model (Table S4)

Two models were retained by the model selection procedure when including relatedness either as a categorical variable or as a continuous variable (Table S4). Both models gave similar qualitative results regarding fixed effects. The height of the focal plant did not depend on relatedness among plants (Table 3, Figure 3). It was not smaller in kin groups (mean _kin_ = 127 cm, mean _non kin_ = 126 cm; *p* = .781) and did not decrease with increasing genetic similarity (*b* = −.164, *p* = .756). In the best model, focal height depended on the focal genotype and the neighbor genotype. For both proxies of relatedness, the proportion of variation in plant height explained by the focal genotype was equal to 35.4% for the first block and 27.8% for the second block. Those explained by the neighbor genotype was equal to 3.8% for the first block and 3.0% for the second block. The genetic covariance between the direct genetic effect of the focal genotype and the indirect genetic effect of this genotype as a neighbor on plant height was not different from zero (Table S4). In contrast to the best model, the second model did not include the effect of the genes of neighbors.

**Table 3:** Results from GLMMs of focal plant height to relatedness

Source of variation	With co‐ancestry			With genetic similarity		
	Df	Wald F	*p*‐value	Df	Wald F	*p*‐value
Intercept	54.8	9,388.800	**<.001**	54.7	9,396.000	**<.001**
Block	53.8	0.009	.925	53.6	0.009	.925
Seed mass	346.7	5.374	**.021**	346.8	5.375	**.021**
Relatedness	294.7	0.078	.781	305.8	0.097	.756

Note

Relatedness was included either as a categorical variable “co‐ancestry” with two classes (kin and nonkin) or as a continuous variable measuring genetic similarity (proportion of shared alleles between the focal genotype and the neighbor genotype). Effects were tested using incremental Wald *F* tests with a 5% significance level after fitting the best model for random‐effect factors (see text for details on model construction and Table S4). The degree of freedom of the numerator was equal to 1 for all terms. We only report the degree of freedom of the denominator. *p*‐Values <.05 are in bold.

### 3.4 Relationship between focal height and inclusive fitness

**Figure 4:**
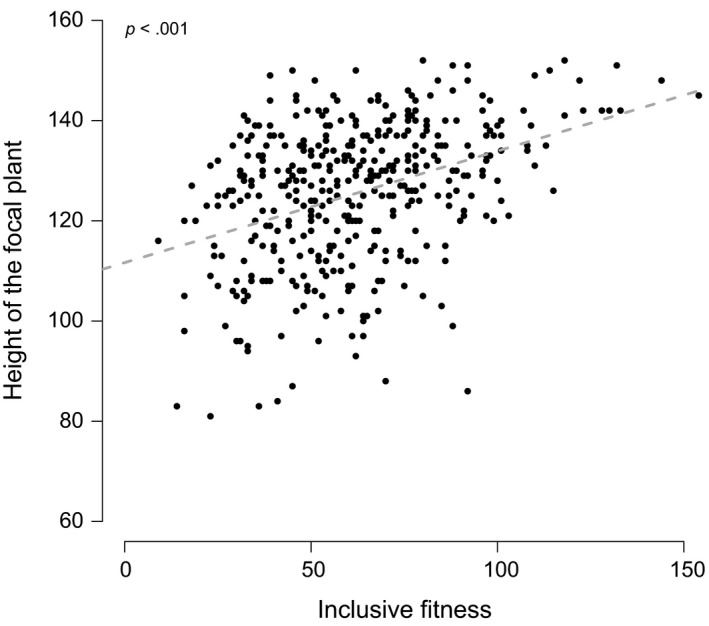
Relationship between the height of the focal plant and inclusive fitness. Dots represent observed values. The linear relationship is plotted according to the best model (Table S5)

Focal height and inclusive fitness were positively correlated (*b* = .223, *p* < .001, Figure 4), after accounting for direct genetic effect of the focal genotype and the indirect genetic effect of this genotype as a neighbor on focal height (Table 4). The genetic covariance between DGE and IGE was not different from zero (Table S5).

**Table 4:** Results from GLMMs of focal plant height to inclusive fitness

Source of variation	Df	Wald F	*p*‐value
Intercept	38.0	10,100	**<.001**
Block	3.4	<0.001	.999
Seed mass	333.3	5.293	**.022**
Inclusive fitness	370.0	103.100	**<0.001**

Note

Effects were tested using incremental Wald *F* tests with a 5% significance level after fitting the best model for random‐effect factors (see text for details on model construction and Table S5). The degree of freedom of the numerator was equal to 1 for all terms. We only report the degree of freedom of the denominator. *p*‐Values <.05 are in bold.

## 4 DISCUSSION

Although preferential helping to relatives has received increased attention in plants in recent decades (File et al., 2012), its role in driving plant–plant interactions in cultivated species remains poorly studied. Still, this might have important implications in agriculture, whenever plants direct helping behaviors preferentially toward relatives and thus experience stronger competition when grown with nonkin, lowering yield of variety mixtures. To our knowledge, evidence for preferential helping to relatives has not been documented in crops. Indeed, the very few studies that have addressed this issue in cultivated species only documented the existence of phenotypic plasticity in response to genetic relatedness to neighbors, but did not measure seed production (Fang et al., 2013; Murphy, Van Acker, Rajcan, & Swanton, 2017; Zhang, Liu, Tian, Xu, & Ouyang, 2016). Yet, many studies in wild plant species have shown that phenotypic plasticity in response to relatedness to neighbors did not necessarily result into higher yield of kin groups (Cheplick & Kane, 2004; Crepy & Casal, 2015; Dudley & File, 2007; Milla et al., 2009). This highlights the need to estimate seed production to test for preferential helping to relatives in crops.

Here, we found that yield did not depend upon relatedness of interacting traditional varieties of durum wheat. Such nonsignificant effect could not be explained by the fact that competition for resources did not occur in our experimental setting. Indeed, plants grown with four neighbors as in our kin experiment had lower fitness and produced less biomass and fewer tillers than plants grown alone, a pattern consistent with wheat response to competition (Donald, 1963; Jennings & Aquino, 1968). This rather suggests that the overall outcome of competition among plants results from different mechanisms that can co‐occur (File et al., 2012), which translate into no net negative or positive effects of competitive interactions on yield. The lack of a general trend from studies investigating competitive interactions among kin and nonkin plants might support this view. While some studies have reported better performance of kin groups (Biernaskie, 2011; Donohue, 2003; Simonsen, Chow, & Stinchcombe, 2014; Tonsor, 1989), others have reported the opposite result (Milla et al., 2009) or no significant trend (Cheplick & Kane, 2004; Dudley & File, 2007; Lepik, Abakumova, Zobel, & Semchenko, 2012; Milla, del Burgo, Escudero, & Iriondo, 2012; Monzeglio & Stoll, 2008). As pointed out by Masclaux et al. (2010), such discrepancies among studies may be due to the existence of direct genetic effects on social traits involved in resource competition, which might lead to among‐genotype differences in the ability to capture resources that do not depend on relatedness. Indeed, if a few genotypes have a higher ability to capture resources because of their genes, these genotypes could lower growth and seed production of other neighbor's genotypes in nonkin groups. This could result in lower seed production in nonkin groups, a pattern consistent with preferential helping of relatives, leading to erroneous interpretations. Such among‐genotype differences are largely ignored in studies investigating preferential helping of relatives, although they are well documented in plants (Biernaskie, 2011; Cahill, Kembel, & Gustafson, 2005; Masclaux et al., 2010; Simonsen et al., 2014). To that respect, IGE models, such as those used here, represent a valuable approach for future studies investigating phenotypic plasticity to relatedness and its consequences on crop yield.

Testing experimentally for preferential help toward relatives remains challenging in plants, where little is known about the traits that are potentially involved in helping. Plant height, a key trait driving interactions among plants, is a good candidate (Anten & Vermeulen, 2016; Dudley et al., 2013), the short stature phenotype being an altruistic behavior for species competing for light. In our experiment, plants were regularly watered and fertilized to limit plant competition for water and nutrients. Therefore, light was likely to be the most limiting factor driving competitive interactions among individuals. Under the kin selection hypothesis involving plasticity in height, we thus hypothesized that plants would adjust their growth toward reduced stem elongation when competing with relatives, resulting in decreasing mean allocation to height with relatedness. Our results do not support this. Kin groups of durum wheat were not shorter than nonkin groups, and there was no significant relationship between mean height of the group and genetic similarity in our experimental setting. Similarly, when accounting for the direct effects of genes of the focal plant and genes of its neighbors on the focal height, we did not detect any evidence of differential stem elongation according to relatedness among plants. Durum wheat plants growing in the vicinity of kin were not shorter than those competing with nonkin, and the height of the focal plant did not decrease with increasing relatedness among plants. Nor do our results support the resource‐use complementarity hypothesis involving a plastic response on height, which predicted increased stem elongation in response to stronger competition for light among more phenotypically similar plants. The very few studies that have documented phenotypic plasticity in plant height in response to kin and nonkin neighbors were performed exclusively on wild species (Biernaskie, 2011; Donohue, 2003; Murphy & Dudley, 2009; Willson et al., 1987), and none of them documented reduced plant height in kin groups. Indeed, some studies also reported no significant effect of relatedness on plant height (Biernaskie, 2011; Donohue, 2003; Willson et al., 1987), while others documented increased plant height in kin groups (Biernaskie, 2011; Donohue, 2003; Murphy & Dudley, 2009; Willson et al., 1987), a pattern expected under the resource‐use complementarity hypothesis. Interestingly, we found that the genotype of the neighbor only explained a very small proportion of the variation in focal height (less than 4%) and was not even included as an explanatory variable in the second best model retained by the selection model procedure. Phenotypic plasticity in plant height in response to neighbor genotypes was thus very limited, if any, in our set of durum wheat genotypes. These results are consistent with the general view that human selection in crops has acted to attenuate shade‐avoidance responses such as increased stem elongation in response to competition for light, during the domestication process and plant breeding (Kebrom & Brutnell, 2007). Future research on preferential helping to relatives in crops might thus target social traits for which a tragedy of the commons has been described when plants compete for resources other than light, such as root traits in the case of competition for soil resources (Anten & Vermeulen, 2016). In contrast to our working hypothesis, we did not find a negative relationship between plant height and inclusive fitness, after accounting for DGE and IGE effects on height. On the contrary, we showed that plant height and inclusive fitness were positively correlated. Because plant height did not show any plastic response to relatedness, such pattern might result from competitive interactions among plants that are not mediated by relatedness among competing plants. Further studies are needed to test whether such a positive covariation has a genetic basis or might result from among‐pot differences in environmental conditions.

Preferential helping to relatives relies on the existence of kin recognition systems (Hamilton, 1964b; Rousset & Roze, 2007), which remains controversial in plants since the pioneer work of Dudley and File (2007) (see for instance Klemens, 2008; Till‐Bottraud & de Villemereuil, 2016). Genetically based kin recognition, where helping occurs between individuals that share alleles at matching loci (Grafen, 1990), has been demonstrated for different taxa including microorganisms and animals (Lehmann & Perrin, 2002). Phenotype matching is part of this mechanism, whenever the phenotypic cue has a genetic basis (Grafen, 1990; Rousset & Roze, 2007). In plants, evidence for the existence of matching loci is still lacking. Genes with significant changes in expression in nonkin versus kin secretions of roots have been identified in *Arabidopsis thaliana* (Biedrzycki, Venkatachalam, & Bais, 2011). Manipulating the genetic composition of groups at those loci to quantify yield in response to relatedness at these loci would be the next necessary step to bring convincing evidence of kin recognition systems in this species. Theoretical work has shown that the conditions allowing for the evolution and maintenance of genetically based kin recognition are often very restrictive (Grafen, 1990; Lehmann & Perrin, 2002; Penn & Frommen, 2010; Rousset & Roze, 2007). In crops, recurrent bottlenecks during domestication and subsequent agronomic improvement have led to major losses of genetic diversity in elite varieties grown in modern agrosystems. For instance, the level of genetic diversity observed in elite varieties of durum wheat corresponded to a 78% reduction of the effective population size of old varieties, consecutive to the selection of semi‐dwarf varieties and a change in cultivation practices since the Green Revolution (Thuillet et al., 2005). Thus, we expect genetic diversity at kin recognition systems in cultivated species to be much lower, if any, in elite varieties (Murphy, Swanton, Van Acker, & Dudley, 2017). Still, if polymorphism has been maintained, genetically based kin recognition may explain conflicting results among studies of variety mixtures within species (Reiss & Drinkwater, 2018; Smithson & Lenné, 1996) and the outcome of competition among varieties depending upon their genetic similarity at the kin recognition loci. Here, we did not find evidence that plants could direct helping toward relatives in durum wheat landraces from traditional agrosystems, where genetic diversity is still present at the field and individual levels (this study, Muller et al unpublished). Whether such ability does exist in wild progenitors of cultivated plants and may have been lost during domestication is unknown. This issue warrants further investigation provided that genetic diversity from the wild pool is used in breeding programs and may thus affect interactions among plants grown in mixtures.

Whether kin recognition systems do exist in some crops and can affect the outcome of competition in variety mixtures needs to be investigated more broadly and further tested under field conditions, with a special emphasis on species grown in monogenotypic cultures of pure lines such as wheat, rice, and barley, for which variety mixtures are the most straightforward way to increase within‐crop genetic diversity. Overall, our study calls for extending evolutionary approaches to address pressing agricultural issues.

## Data Availability

Data for this study are available at the INRA data repository: https://doi.org/10.15454/20RKZ6 (Fréville, 2019).
