# Supplementary material for: Preferential helping to relatives: A potential mechanism responsible for lower yield of crop variety mixtures?
Source: Evol Appl. 2019 Aug 1;12(9):1837–49. doi: 10.1111/eva.12842 (PMC6752151; doi:10.1111/eva.12842)
Supplement: Supplementary file 1 [file EVA-12-1837-s001.pdf]

TABLE S1. Description of the 26 microsatellite nuclear loci and summary statistics computed over the 52 maternal plants used in the kin experiment. The SSR markers included gwm (Röder et al. 1998), cfa and gpw (Sourdille et al. 2003) and wmc (Somers et al. 2004) wheat SSRs. N_A : number of alleles, H_E : gene diversity corrected for sample size (Nei 1978).

Locus	Primer sequence (5'-3')	Primer volume (10 pM)	Multiplex group	5'-label	Annealing (°C)	Allelic range (bp)	N_A	H_E
Xgwm234	F: CTCATTGGGGTGTGTACGTG<br>R: GAGTCCTGATGTGAAGCTGTTG	0.2 µl<br>0.4 µl	1	6-FAM	55	216-252	7	0.651
Xgpw3029	F: AGCTGATGGAGACAACCTTT<br>R: AGGTAATTCTTCCCCCTCCG	0.2 µl<br>0.4 µl	2	HEX	57	233-237	3	0.517
Xgwm372	F: AATAGAGCCCTGGGACTGGG<br>R: GAAGGACGACATTCCACCTG	0.5 µl<br>1.0 µl	3	TAMRA	60	305-317	4	0.184
Xgpw5210	F: CAGCGGACCTAAGCATCATA<br>R: CGCTAAAGATGAAGTGAAGACA	0.5 µl<br>1.0 µl	4	TAMRA	58	128-139	4	0.638
Xwmc25	F: TCTGGCCAGGATCAATATTACT<br>R: TAAGATACATAGATCCAACACC	0.5 µl<br>1.0 µl	5	TAMRA	51	183-191	4	0.509
Xcfa2141	F: GAATGGAAGGCGGACATAGA<br>R: GCCTCCACAACAGCCATAAT	0.2 µl<br>0.4 µl	6	6-FAM	60	245-265	7	0.719
Xgpw2331	F: GCGGGCTCAATATTGCTAGT<br>R: GCATGGCTGAGGCTCAAGTA	0.3 µl<br>0.6 µl	6	TAMRA	60	217-225	5	0.652
Xgwm389	F: ATCATGTCGATCTCCTTGACG<br>R: TGCCATGCACATTAGCAGAT	0.3 µl<br>0.6 µl	7	6-FAM	60	115-123	4	0.591
Xgwm610	F: CTGCCTTCTCCATGGTTTGT<br>R: ATTGGCCAAAGGTTATGAAGG	0.4 µl<br>0.8 µl	7	HEX	60	155-167	6	0.539
Xgpw2276	F: ATAGGGTTCTTCTGTGCCCC<br>R: ACCCACAGTTGAACTTGGG	0.4 µl<br>0.8 µl	7	TAMRA	60	198-207	3	0.146

Xgpw2279	F: GGTGTTCTCGGTCGTCAGAT<br>R: CTCACCCAACCAATGCCTAT	0.4 µl<br>0.8 µl	8	HEX	60	211-228	6	0.671
Xgpw4078	F: TTAACTAATGCGGTTATTGGCA<br>R: CCACGGAGCCTTTTATTTATT	0.5 µl<br>1.0 µl	8	TAMRA	60	252-256	3	0.467
Xgwm46	F: GCACGTGAATGGATTGGAC<br>R: TGACCCAATAGTGGTGGTCA	0.2 µl<br>0.4 µl	8	6-FAM	60	172-185	6	0.571
Xgpw2239	F: CAACCATATGCCCAGGAGAC<br>R: TGTTGCTGTCTGAAACAGGG	0.45 µl<br>0.90 µl	9	TAMRA	60	193-195	2	0.206
Xgpw7148	F: GCACACAACGACACTTGCTT<br>R: GCTTAGCTGCTTGCTTTGTG	0.3 µl<br>0.6 µl	9	HEX	60	84-103	5	0.592
Xgpw4004	F: CGCCTCGGATTCTATTCTTG<br>R: CTTACTGCGGCCTTGAGTTG	0.45 µl<br>0.90 µl	9	TAMRA	60	224-236	4	0.335
Xgpw3256	F: TCATCAACGACAACGGGAC<br>R: GAGCTGCCTCATGTCGCTT	0.3 µl<br>0.6 µl	10	6-FAM	60	161-165	3	0.499
Xgpw4103	F: CTAGCCCAGTGTCAGGCTTC<br>R: GATGGAATGCAACCAGGC	0.3 µl<br>0.6 µl	10	TAMRA	60	275-289	5	0.658
Xgpw7101	F: CCTGGTATGTATGGTAGAGCCC<br>R: ACCGCAGTGGCTGAAATC	0.3 µl<br>0.6 µl	10	HEX	60	140-142	2	0.176
Xgpw2302	F: GCTTCACATCATAGTGTGGATAAGA<br>R: AAGCACCTCCCATGCATATC	0.25 µl<br>0.50 µl	11	HEX	60	175-189	5	0.488
Xgpw4082	F: CTTTCTTTCCCCTCCTGTCC<br>R: ATCATCACAAATGCAGCGAG	0.45 µl<br>0.90 µl	11	TAMRA	60	229-233	3	0.247
Xwmc231	F: CATGGCGAGGAGCTCGGTGGTC<br>R: GTGGAGCACAGGCGGAGCAAGG	0.35 µl<br>0.70 µl	11	HEX	60	249-255	2	0.482
Xgpw2071	F: GTCCTTCTTGCAATGGGCTA<br>R: TGTTCATGGTTGAGTGCAAAG	0.4 µl<br>0.8 µl	12	HEX	60	279-283	3	0.619
Xgpw3094	F: TGGAGTACATGACAACCACCA<br>R: TCCCAAATTACAAAGAGGCG	0.15 µl<br>0.30 µl	12	6-FAM	60	233-235	2	0.430
Xgpw3117	F: AGCAACTGCTACCAGCAA	0.45 µl	12	TAMRA	60	246-248	2	0.143

	R: GTACCGCTGCATGTCATT	0.90 µl						
Xgpw3142	F: GCCTTGCTCCTCTCCTACCT<br>R : GTATCACCCATCGCTGCC	0.2 µl<br>0.4 µl	12	HEX	60	199-215	5	0.233

DNA extraction

DNA was extracted from 200 mg of fresh young leaves with the DNeasy Plant Mini Kit (Qiagen), used according to the manufacturer's instructions, but with the addition of 1% polyvinylpyrrolidone (PVP 40 000) to the AP1 buffer.

Genotyping

Amplification reactions were performed in a final volume of 20 µl in the presence of 50 ng of template DNA, x*2 pmol of the reverse primer and x pmol of the forward primer (see Table), 0.2 mM of each deoxynucleotide, 2 mM MgCl2, and 0.5 unit Taq polymerase (Qiagen). DNA amplification was performed separately for 5 loci, and in duplex, triplex or quadruplex for the other 21 loci, depending of their annealing temperature (see column 'multiplex group'). The forward primer was 50 -labeled with one of the three fluorophores (6FAM, Tamra or HEX). PCR was carried out using a Master-Cycler, ep gradient S thermocycler Eppendorf) as follows: after 5 min at 94°C, 35 cycles were performed with 1 min at 94°C, 1 min at annealing temperature, and 2 min at 72°C, followed by final extension step of 30 min at 72°C. Amplified products were detected on an ABI 3130xl Genetic Analyser (Applied Biosystems, Foster City, CA, USA). Samples were prepared by adding 3 µl of diluted PCR products to 16.5 µl water and 0.5 µl GenSize AMM524 Rox. Analyses were performed using the GENEMAPPER V3.7 software (Applied Biosystems).

References

Nei M (1978) Estimation of average heterozygosity and genetic distance for small number of individuals. Genetics 89: 583-590.

Röder M, Korzun V, Wendhake K, Plaschke J, Tixier M, Leroy P, Ganal M (1998) A microsatellite map of wheat. Genome 149: 2007–2023.

Somers D, Isaac P, Edwards K (2004) A high-density microsatellite consensus map for bread wheat (*Triticum aestivum* L.). Theor Appl Genet 109: 1105–1114.

Sourdille P, Cadalen T, Guyomarc'h H, Snape J, Perretant M, Charmet G, Boeuf C, Bernard S, Bernard M (2003) An update of the Courtot X Chinese Spring intervarietal molecular marker linkage map for the QTL detection of agronomic traits in wheat. Theor Appl Genet 106: 530–538.

TABLE S2. Results of model selection for mean height of the group. Relatedness was either included as a categorical variable 'coancestry' with two classes (kin, non kin) or a continuous variable measuring 'genetic similarity' (proportion of shared alleles between the focal genotype and the neighbour genotype). The full model refers to Equation (1) in the main text. Alternative models are listed by increasing ΔAICc values. k: number of fitted parameters, LogL : LogLikelihood of the model, ΔAICc: difference in corrected Akaike's information criteria with the best model, Weight: model weight.

	With coancestry				With genetic similarity			
Model	k	LogL	ΔAICc	Weight	k	LogL	ΔAICc	Weight
Full model	14	-1163.50	0	1.000	13	-1164.01	0	1.000
No uncorrelated error	13	-1172.71	16.286	0.000	12	-1173.34	16.529	0.000
No correlated error	8	-1226.19	112.690	0.000	7	-1226.60	112.558	0.000

TABLE S3. Results of model selection for yield. Relatedness was either included as a categorical variable 'coancestry' with two classes (kin, non kin) or a continuous variable measuring 'genetic similarity' (proportion of shared alleles between the focal genotype and the neighbour genotype). The full model refers to Equation (1) in the main text. Alternative models are listed by increasing ΔAICc values. k: number of fitted parameters, LogL : LogLikelihood of the model, ΔAICc: difference in corrected Akaike's information criteria with the best model, Weight: model weight. Tests of significance of fixed effects gave similar results when fitting the best model and the second best model with ΔAICc < 2.

	With coancestry				With genetic similarity			
Model	k	LogL	ΔAICc	Weight	k	LogL	ΔAICc	Weight
Full model	14	-1807.94	0	0.571	13	-1808.68	0	0.574
No uncorrelated error	13	-1809.30	0.572	0.429	12	-1810.05	0.597	0.426
No correlated error	8	-1873.33	118.042	0.000	7	-1873.92	117.818	0.000

TABLE S4. Results of model selection for the height of the focal plant as a function of relatedness. All models included block, seed mass and relatedness as fixed effects, but differed in their random structure. Random effects included in each model are indicated with a star (*) (σ_{fn}: genetic covariance between the direct genetic effect of the focal genotype and the indirect genetic effect of this genotype as a neighbor; ε_{cor}: spatially correlated environment variance, ε_{uncor}: spatially correlated environment variance). Models are ordered according to increasing ΔAICc values. k: number of fitted parameters, LogL: LogLikelihood of the model, ΔAICc: difference in corrected Akaike's information criteria with the best model, Weight: model weight. The full model M_0 that includes all random-effect factors refers to Equation (2) in the main text. Tests of significance of fixed effects gave similar results when fitting the best model and the second best model with ΔAICc < 2. Relatedness was included as a categorical variable 'coancestry' with two classes (kin, non kin) or a continuous variable 'genetic similarity' (proportion of shared alleles between the focal genotype and the neighbor genotype).

		Random-effect factors					Model selection parameters			
	Model	focal	neighbor	σ_{fn}	ε_{cor}	ε_{uncor}	k	LogL	ΔAICc	Weight
Coancestry	M_4	*	*		*		14	-1227.024	0.000	0.358
	M_7	*			*		13	-1228.455	0.720	0.250
	M_1	*	*	*	*		15	-1227.024	2.153	0.122
	M_3	*	*		*	*	15	-1227.024	2.153	0.122
	M_6	*			*	*	14	-1228.455	2.862	0.086
	M_0	*	*	*	*	*	16	-1227.024	4.317	0.041

	M_5	*	*			*	9	-1235.859	7.064	0.010
	M_8	*				*	8	-1237.501	8.259	0.006
	M_2	*	*	*		*	10	-1235.850	9.147	0.004
Genetic similarity	M_4	*	*		*		13	-1227.808	0.000	0.360
	M_7	*			*		12	-1229.254	0.760	0.246
	M_1	*	*	*	*		14	-1227.807	2.141	0.123
	M_3	*	*		*	*	14	-1227.808	2.143	0.123
	M_6	*			*	*	13	-1229.254	2.892	0.085
	M_0	*	*	*	*	*	15	-1227.807	4.294	0.042
	M_5	*	*			*	8	-1236.616	7.062	0.011
	M_8	*				*	7	-1238.279	8.308	0.006
	M_2	*	*	*		*	9	-1236.608	9.136	0.004

TABLE S5. Results of model selection for the height of the focal plant as a function of inclusive fitness. All models included block, seed mass and inclusive fitness as fixed effects, but differed in their random structure. Random effects included in each model are indicated with a star (*) (σ_{fn}: genetic covariance between the direct genetic effect of the focal genotype and the indirect genetic effect of this genotype as a neighbor; ε_{cor}: spatially correlated environment variance, ε_{uncor}: spatially correlated environment variance). Models are ordered according to increasing ΔAICc values. k: number of fitted parameters, LogL: LogLikelihood of the model, ΔAICc: difference in corrected Akaike's information criteria with the best model, Weight: model weight. The full model M_0 that includes all random-effect factors refers to Equation (2) in the main text.

	Random-effect factors					Model selection parameters			
Model	focal	neighbor	σ_{fn}	ε_{cor}	ε_{uncor}	k	LogL	ΔAICc	Weight
M_3	*	*		*	*	14	-1185.309	0.000	0.699
M_0	*	*	*	*	*	15	-1185.306	2.147	0.239
M_7	*			*		12	-1190.250	5.607	0.042
M_4	*	*		*		13	-1190.250	7.739	0.015
M_1	*	*	*	*		14	-1190.249	9.880	0.005
M_8	*				*	7	-1201.865	18.335	0.000
M_5	*	*			*	8	-1201.865	20.415	0.000
M_2	*	*	*		*	9	-1201.862	22.499	0.000
M_6	*			*	*	13	-1285.630	198.492	0.000
